# Dealing with iron metabolism in rice: from breeding for stress tolerance to biofortification

**DOI:** 10.1590/1678-4685-GMB-2016-0036

**Published:** 2017-03-16

**Authors:** Railson Schreinert dos Santos, Artur Teixeira de Araujo, Camila Pegoraro, Antonio Costa de Oliveira

**Affiliations:** 1 Plant Genomics and Breeding Center (CGF), Universidade Federal de Pelotas, Pelotas, RS, Brazil.; 2 Technology Development Center (CDTec), Universidade Federal de Pelotas, Pelotas, RS, Brazil.

**Keywords:** iron toxicity, mineral malnutrition, Fe-enrichment, Quantitative Trait Loci

## Abstract

Iron is a well-known metal. Used by humankind since ancient times in many different ways, this element is present in all living organisms, where, unfortunately, it represents a two-way problem. Being an essential block in the composition of different proteins and metabolic pathways, iron is a vital component for animals and plants. That is why iron deficiency has a severe impact on the lives of different organisms, including humans, becoming a major concern, especially in developing countries where access to adequate nutrition is still difficult. On the other hand, this metal is also capable of causing damage when present in excess, becoming toxic to cells and affecting the whole organism. Because of its importance, iron absorption, transport and storage mechanisms have been extensively investigated in order to design alternatives that may solve this problem. As the understanding of the strategies that plants use to control iron homeostasis is an important step in the generation of improved plants that meet both human agricultural and nutritional needs, here we discuss some of the most important points about this topic.

## Introduction

Iron is the fourth most abundant element in the earth’s crust, where ferric iron (Fe^3+^) and ferrous iron (Fe^2+^) are the most common forms (Hori *et al*., 2015). While Fe^3+^ is insoluble and its uptake is difficult, Fe^2+^ is soluble and readily available to plants. When the soil is aerated and in alkaline pH, Fe is oxidized as insoluble iron oxides, but in flooded soils, which are in anaerobic conditions, pH decreases and there is a reduction of Fe^3+^ to Fe^2+^ (Morrissey and Guerinot, 2009). This event is responsible for the low availability of Fe in upland soils and for its high availability in flooded soils.

Fe is an essential micronutrient for both animals and plants. In mammals iron is part of the structure of a diversity of proteins (hemoglobin, myoglobin, cytochromes, flavoproteins, heme-flavoproteins, transferrin, lactoferrin, ferritin, hemosiderin, sulfur, non-heme enzymes) (Institute of Medicine, 2001). In plants, Fe serves as a component of many vital enzymes such as cytochromes of the electron transport chain, acting in photosynthesis and in the electron transfer (through Fe-S clusters), in respiration, and other important metabolic pathways (Briat and Lobreaux, 1997; Kobayashi and Nishizawa, 2012; Rout and Sahoo, 2015). It also participates in the Fenton reaction catalyzing the generation of hydroxyl radicals (OH), and reactive oxygen species (ROS) that can cause irreversible damage to the cell (Wu *et al*., 2014). Thus, Fe stress can be caused either by deficiency as well as by excess (Connolly and Guerinot, 2002).

Iron deficiency can cause alterations in root morphology (Morrissey and Guerinot, 2009; Giehl *et al*., 2012; Gruber *et al*., 2013) and chlorosis of young leaves, therefore reducing yield (Kobayashi and Nishizawa, 2014). To prevent the shortage of this element, plants have developed two different absorption strategies: strategy I, which is used by higher plants, except for members of the Poaceae family. In this strategy the enzyme H^+^ ATPase (AHA) mediates the release of hydrons from the roots to the rhizosphere, increasing the solubility of Fe^3+^, and the Phenolics Efflux Zero 1 (PEZ1) transports phenolics, such as protocatechuic acid, making it possible to take up and use apoplastic precipitated Fe (Ishimaru *et al*., 2011; Rodríguez-Celma and Schmidt, 2013). A comparison between two model species, Arabidopsis and *Medicago truncatula*, showed further evidence that the production and secretion of phenolic compounds is critical for the uptake of iron from sources with low bioavailability, but dispensable under conditions where iron is readily available (Rodríguez-Celma *et al*., 2013; Rodríguez-Celma and Schmidt, 2013).

Also, in strategy I, Ferric Reductase Oxidase (FRO2) mediates the Fe^3+^ reduction to Fe^2+^, and Iron Regulated Transporter1 (IRT1) is responsible for Fe^2+^ absorption by the roots (Connolly and Guerinot, 2002; Kobayashi and Nishizawa, 2012).

Strategy II, which is specific of grasses, is based on biosynthesis and secretion of compounds called phytosiderophores (PS), which are results of the action of nicotianamine synthase (NAS), nicotianamine aminotransferase (NAAT) and deoxymugineic acid synthase (DMAS) (Shojima *et al*., 1990). TOM1/OsZIFL4, which belongs to the major facilitator superfamily (MFS) (Pao *et al*., 1998), is involved in siderophore export necessary in Fe acquisition (Furrer *et al*., 2002). These PSs can bind to Fe^3+^ forming the soluble complex Fe(III)-PS, and these complexes in the rhizosphere can be taken up into root cells through the action of YELLOW STRIPE-LIKE PROTEINS (YSLs) (Inoue *et al*., 2009; Lee *et al*., 2009a; Nozoye *et al*., 2011). In rice there are 18 YS1-like (OsYSL) genes, and OsYSL15 transports Fe(II)-PS and it is likely more relevant for Fe(III)-PS (Romheld and Marschner, 1986; Curie *et al*., 2001; Inoue *et al*., 2009; Lee *et al*., 2009a). Rice (*Oryza sativa* L.) uses strategy II, but is also able to absorb Fe^2+^ directly from the rhizosphere through IRT1 (Zaharieva and Römheld, 2000; Bughio *et al*., 2002; Ishimaru *et al*., 2006; Kobayashi and Nishizawa, 2014).

The high level of Fe^2+^ found in some flooded soils can be toxic to plants (Mongon *et al*., 2014). The toxicity caused by excessive Fe can occur directly and indirectly. The direct toxicity occurs when there is too much absorption and excessive accumulation of this element in tissues followed by the appearance of brown-dark spots in the leaves (leaf-bronzing) (Becker and Asch, 2005; Morrissey and Guerinot, 2009). The indirect damage is caused by the prevention of the uptake, transport and utilization of other nutrients (e.g.: P, K, Ca, Mg, Mn, and Zn) due to the iron plaque that forms when Fe^3+^ is deposited in the apoplast of rice roots (Sahrawat, 2004; Zhang *et al*., 2014). Both situations affect plant growth, development and productivity, leading to significant yield losses. To adapt to this condition, rice plants have developed different mechanisms of tolerance (Type I, Type II and Type III) that are based on specific forms of use, exclusion and storage of iron. In Type I there is an oxidation and precipitation of Fe^2+^ on the root surface, while in Type II the storage occurs in a less reactive form, in ferritin protein. Type III mechanism is based on tolerance to the ROS formed in Fenton’s reaction (Wu *et al*., 2014). Another thing that can occur is the annulment of the absorbed Fe^2+^ by its storage in old or less active leaves or exclusion via symplast (Becker and Asch, 2005).

Physiological disorders caused by Fe excess are common in cultivated rice in the regions of Africa, Asia and South America (Shahid *et al*., 2014). However, despite the high amount of Fe in the soil, which can even be toxic to the plant, little is accumulated in rice grains. In addition, the accumulation of iron in the grain occurs in the outermost layers being lost during the industrial processing (Doesthale *et al*., 1979; Sperotto *et al*., 2012). Thus, rice contributes very little to meet the need of Fe intake in the human diet, not being an effective way of preventing anemia.

More than two billion people worldwide suffer from anemia, and more than 50% of these cases are caused by Fe deficiency (Arcanjo *et al*., 2013). The Fe-deficiency anemia (IDA) affects more dramatically the continents of Africa and Asia, where IDA is a major public health problem, prevalent in young women and children (Moretti *et al*., 2006; Visser and Herselman, 2013), since it is responsible for the death of almost one million individuals per year (Aung *et al*., 2013).

Biofortification is an interesting strategy to solve the problem of IDA, especially for people who cannot change their eating habits due to financial, cultural or religious issues. In this sense, not only increasing the amount of iron in grains, but also decreasing the content of inhibitors of Fe absorption commonly found in plants can improve the diets (Lucca *et al*., 2001; Raboy, 2002; Schuler and Bauer, 2012). In addition, biofortification is a sustainable strategy. In this sense rice can be the ideal species for biofortification since it is a staple food that is especially important for developing countries, where IDA is even more severe. Also, rice is grown in flooded soils, where Fe availability is higher (Becker and Asch, 2005), and has its mechanisms of absorption, translocation and homeostasis Fe better understood than most of the species (Masuda *et al*., 2012).

Global rice production is 741 million tons at approximately 165 million hectares. Rice is not only the second most cultivated cereal in the world, with important social and economic function, but is also an ideal model for functional genomics studies in monocots (FAOSTAT, 2015; Yao *et al*., 2015). The availability of different rice genomes of different subspecies has enabled the study of many genes and metabolic pathways (Goff *et al*., 2002; IRGSP - International Rice Genome Sequencing Project, 2005).

Considering the importance of rice in nutrition and economy as well as the impact of iron deficiency and excess in the life of plants and animals, in this review we will discuss the highlights of the uptake pathways, translocation, homeostasis and Fe accumulation in the grain. Understanding these points is essential both to solve the problem of sensitivity to high levels of Fe as to allow Fe-biofortification.

## Identifying regulatory pathways

**Figure 1:**
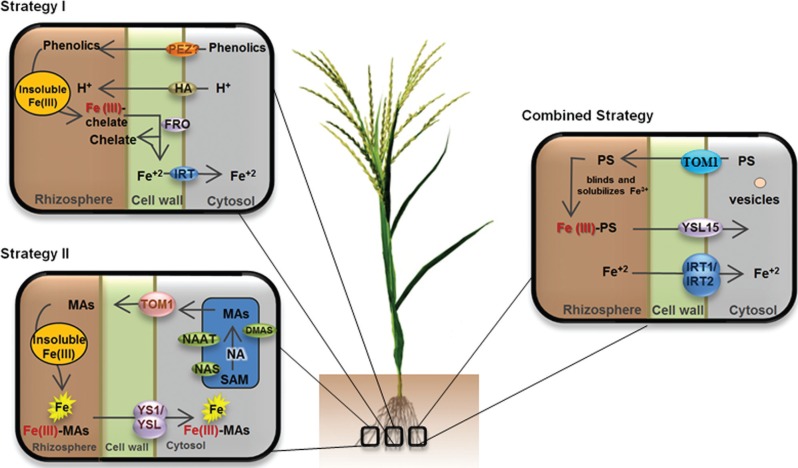
Absorption and translocation of iron in rice. Adapted from Palmer and Guerinot (2009); Kobayashi and Nishizawa (2012); Bashir *et al*. (2013a).

According to the availability of Fe in the soil, plants have developed mechanisms to control and regulate the absorption, translocation and subcellular storage of this mineral. Classical studies associated with the emergence of modern and advanced tools of genomics, transcriptomics and proteomics have enabled in-depth understanding of homeostasis of Fe in plants (Kobayashi and Nishizawa, 2012). The uptake of Fe occurs by using strategy I or reduction (non Poaceae), strategy II or chelation (Poaceae), and a combination of strategies I and II (rice) (Figure 1) (Ishimaru *et al*., 2006; Zhang *et al*., 2012; Yang *et al*., 2013; Ricachenevsky and Sperotto, 2014; Finatto *et al*., 2015). The key genes involved in strategy I are AHA2 (protonation of the rhizosphere), FRO2 (reduction of Fe^3+^ to Fe^2+^), and IRT1 (Fe^2+^ transport into the root) (Kim and Guerinot, 2007; Hindt and Guerinot, 2012).

In *Arabidopsis thaliana* (L.) Heynh there are eight homologues of *FRO* (*AtFRO1* to *AtFRO8*), while in *O. sativa* there are only two (*OsFRO1* and *OsFRO2*) (Victoria *et al*., 2012). The gene *IRT* presents 15 homologues in *A. thaliana*, (*AtIRT1*, *AtIRT2*, *AtIRT3*, *AtZIP1* to *AtZIP12*) and 11 in *O. sativa* (*OsIRT1*, *OsIRT2*, *OsZIP1* to *OsZIP10*) (Ishimaru 2005; Ishimaru *et al*., 2006; Kim and Guerinot 2007). *A. thaliana* presents 12 homologues of the gene *AHA* (*AtAHA1* to *AtAHA12*) (Santi and Schmidt, 2009) and *O. sativa* ten (*OsA1* to *OsA10*) (Zhu *et al*., 2009; Li *et al*., 2015). Not all members of FRO, ZIP and AHA families are directly involved with Fe capture (Michelet and Boutry, 1995; Bernal *et al*., 2012; Milner *et al*., 2013).

In conditions of Fe deficiency there is an induction of *IRT1*, *FRO2* and several *AHAs* (Colangelo and Guerinot, 2004; Santi and Schmidt, 2009; Hindt and Guerinot, 2012). Studies conducted in *A. thaliana* demonstrate that the low availability of Fe leads to the induction of transcription factor (TF) FER-like iron deficiency-induced transcription factor (*FIT*) which regulates *AtFRO2* at the level of mRNA accumulation and *AtIRT1* at the level of both mRNA and protein accumulation (Eide *et al*., 1996; Vert *et al*., 2002; Colangelo and Guerinot, 2004). The co-expression of *FIT* with other TFs of the *Basic helix-loop-helix* (*AtbHLH38/39*) family directly regulates the expression of *IRT1* and *FRO2*, increasing iron accumulation (Yuan *et al*., 2008; Hindt and Guerinot, 2012). There are no orthologs of *FIT* in rice, but *AtbHLH38/39* are similar to *OsIRO2* (Hindt and Guerinot 2012) that regulates genes related to transport of Fe(III)-PS, but does not regulate *OsIRT1* (Ogo *et al*., 2007).

The *FIT* gene is regulated by signaling molecules such as auxin and ethylene, synthesized in conditions of iron deficiency. In Arabidopsis the lack of Fe induces an increase in auxin synthesis, resulting in increased expression of the genes *FIT* and *FRO2* (Chen *et al*., 2010). Similarly to what happens to auxin, an increase in ethylene synthesis is also noticed under these conditions, an event that cause the upregulation of *FIT* (Lucena *et al*., 2006) and therefore of *FRO* and *IRT*. FIT interacts with the TFs *Ethylene insensitive 3* (*AtEIN3*) and *Ethylene insensitive 3–like1* (*AtEIL1*) emphasizing the importance of ethylene signaling in response to Fe deficiency (Lingam *et al*., 2011). It is interesting to note that there is a plethora of bHLH genes involved in iron uptake regulation and extensive additional information is available (Bashir *et al*., 2010; Zheng *et al*., 2010; Zhao *et al*., 2014; Li *et al*., 2016).

Just as auxin and ethylene, nitric oxide (NO) has its synthesis increased in conditions of Fe deficiency. NO acts as a positive regulator of genes whose products act on Fe uptake (Hindt and Guerinot, 2012). Conversely, under conditions of Fe excess, three *ZIP* genes and *OsFRO2* are induced in rice (Finatto *et al*., 2015).

In Arabidopsis the *Popeye* (*AtPYE*) and *Brutus* (*AtBTS*) genes are, respectively, a TF and an E3 ubiquitin ligase that also participate in the regulation of Fe absorption. These proteins act in sensitizing the root response to the availability of Fe, regulating Fe homeostasis (Long *et al*., 2010). In rice, the genes *OsIRO3* (Zheng *et al*., 2010) and *OsHRZ1/OsHRZ2* (Kobayashi *et al*., 2013), have been identified. *IRO3* is an ortholog of *AtPYE*, and *HZR1* and *HZR2* are orthologs of *AtBTS*.

Strategy II (Figure 1) includes the participation of genes that act in the cycle of PSs precursors - METHIONINE and S-ADENOSYL-L-METHIONINE (*5’-*methylthioadenosine nucleosidase – *MTN*, Methylthioribose kinase – *MTK*, Methylthioribose-1-phosphate isomerase – *IDI2* and dehydrase enolase phosphatase – *DEP,* s-adenosyl-l-methionine synthetase – *SAMS*) (Kobayashi *et al*., 2005; Suzuki *et al*., 2006), in the synthesis of PSs (*NAS*, *NAAT*, *DMAS,* Dioxygenases – *IDS2/IDS3*) (Nakanishi *et al*., 2000; Kobayashi and Nishizawa, 2012), binding of PSs to Fe(III) (Nozoye *et al*., 2011), and in the transport of the complex Fe(III)-PSs into the root (*YS1* and *YSL*) (Curie *et al*., 2001; Inoue *et al*., 2009; Lee *et al*., 2009a; Kobayashi and Nishizawa, 2012). Four homologues of the gene *NAS* are present in arabidopsis (*AtNAS1*, *AtNAS2*, *AtNAS3* and *AtNAS4*) and three in rice (*OsNAS1*, *OsNAS2* and *OsNAS3*) (Victoria *et al*., 2012). Six homologues of the *NAAT* gene (*OsNAAT1* to *OsNAAT6*), and only one *DMAS* gene (*OsDMAS1*) are present in rice (Bashir *et al*., 2006; Widodo *et al*., 2010). For gene *YSL,* eight homologues were identified in Arabidopsis (*AtYSL1* to *AtYSL8*) and 18 in rice (*OsYSL1* to *YSL18*) (Victoria *et al*., 2012).

Like as the genes involved in strategy I, genes associated with strategy II are induced in iron deficiency (Ricachenevsky and Sperotto, 2014). The TFs *Iron deficiency responsive element binding factor 1* (*IDEF1* and *IDEF2)* and *Iron regulated basic helix-loop-helix* (*IRO2*) have been identified as regulators of key genes that control Fe uptake, including the synthesis of PSs in rice (Itai *et al*., 2013). Under Fe deficiency the *OsIDEF1* upregulates genes whose products act in capture and use of Fe in rice, such as *OsIRO2*, *OsYSL15*, *OsYSL2*, *OsIRT1*, *OsNAS1*, *OsNAS2* and OsNAS3 (Kobayashi *et al*., 2009). The TF *IDEF1* binds to Iron Deficiency-responsive Element 1 (IDE1), while *IDEF2* binds to IDE2, both present in the promoter region of genes associated with Fe deficiency (Kobayashi *et al*., 2007; Ogo *et al*., 2008). Moreover, *OsIRO3* is induced in Fe deficiency and acts as a negative regulator of genes related to this condition in rice (*OsNAS1*, *OsNAS2*, *OsIRO2*, *OsIRT1*, *OsYSL15* and *OsNRAMP1*) (Zheng *et al*., 2010).

In conditions of Fe toxicity the genes *OsNAS1*, *OsNAS2*, *OsYSL15*, *OsYSL16* and *OsNRAMP1* were repressed in rice roots (Quinet *et al*., 2012). In a similar study, Finatto *et al*. (2015) reported the induction of the genes *OsNAAT1*, *OsYSL1* and *OsYSL17* in rice plants grown under excessive Fe.

**Figure 2:**
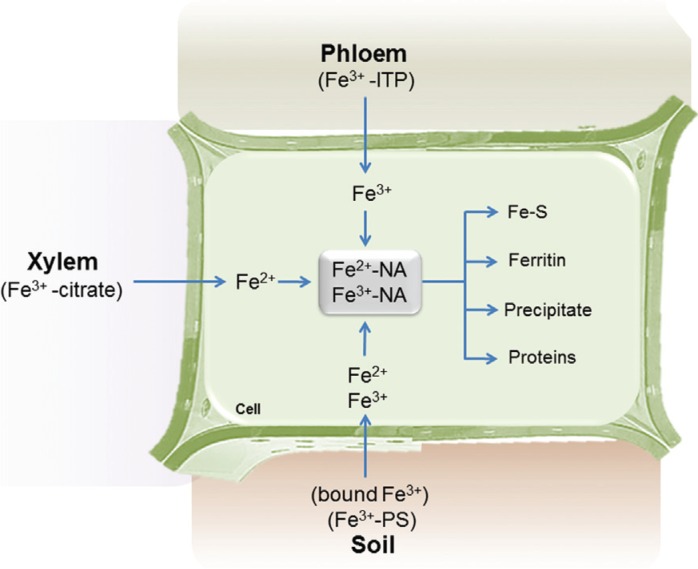
Role of nicotianamine (NA) in iron metabolism in plant cells. Iron can enter the plant cell through various strategies depending on the nature of the iron source. In this context NA is an important chelator that is able to provide iron in a functional form, avoiding precipitation and catalysis. Adapted from Hell and Stephan (2003).

After Fe capture by the roots, this is transported to other organs, a process that involves several steps, passing through symplast, xylem (transpiration stream) and phloem (Kim and Guerinot, 2007). When Fe enters the symplast it is oxidized and ligated to chelating molecules (Miroslav, 1998). Chelators that can bind to Fe are, as shown in Figure 2, citrate, nicotianamine (NA) and mugineic acid (MA) (Kobayashi and Nishizawa, 2012).

It has been proposed that NA facilitates Fe movement in and out of the phloem (through YSLs), while the movement of Fe within the phloem occurs via Iron Transport Proteins (ITP), dehydrins (DHN) that bind Fe^3+^ but not Fe^2+^ (Krüger *et al*., 2002; Hell and Stephan, 2003; Morrissey and Guerinot, 2009). In *A. thaliana* the *Ferric Reductase Defective 3* (*AtFDR3*) encodes a transmembrane protein belonging to the family of Multidrug and toxin efflux transporters (MATE) that facilitates the transport of citrate in the xylem (Durrett *et al*., 2007).

In rice, a citrate transporter called *OsFRDL1* is required for efficient translocation of Fe-citrate complex (Yokosho *et al*., 2009). In rice plants under conditions of Fe excess, the induction of three genes belonging to the MATE family, which may be involved in reducing ROS production in mitochondria, was observed (Finatto *et al*., 2015). Genes belonging to *YSL* and *IRT* families, as well are not only involved in iron uptake, but also in the transport of this element through the plant. Different *YSL* genes transport different complexes. In rice for example, *OsYSL2* transports Fe(II)NA (Koike *et al*., 2004) while *OsYSL15* product transports Fe(III)-DMA (Lee *et al*., 2009a). *OsIRT1* is expressed not only in roots, but also in rice leaves and stems, indicating its participation in the Fe transport over long distances (Narayanan *et al*., 2007).

To be assimilated by the leaves, Fe^3+^ is reduced by FRO enzymes. FRO7 of *A. thaliana* plays a role in chloroplast iron acquisition and is required for efficient photosynthesis in young seedlings and is especially important when plants are under iron-limiting conditions (Jeong *et al*., 2008). The *LeFRO1* of *Lycopersicum esculentum* Mill. (Li *et al*., 2004), *PsFRO1* of *Pisum sativum* (Waters *et al*., 2002) and *AtFRO6* in *A. thaliana* are expressed in the aerial part (Feng *et al*., 2006), indicating their participation in reduction of Fe^3+^. A great diversity of these proteins has been studied, and these are not only involved in iron, but also in copper homeostasis. The diverse roles of the FRO family have recently been reviewed (Jain *et al*., 2014).

After reduction, Fe is transported to other organs of the plant. This transport is performed via the phloem nicotianamine chelator (NA) (Takahashi *et al*., 2003), which is synthesized by the enzyme Nicotianamine synthase (NAS). The Fe transport also occurs through family members of the NATURAL RESISTANCE-ASSOCIATED MACROPHAGE PROTEIN (NRAMP) (Nevo and Nelson, 2006). NRAMP carriers are related to the subcellular transport of Fe and its partitioning in vacuoles and/or plastids (Curie *et al*., 2000). Six *NRAMP* genes were found in Arabidopsis and eight in rice (*OsNRAMP1*-*OsNRAMP8*) (Victoria *et al*., 2012).

In rice, *OsNRAMP1* is expressed mainly in roots, *OsNRAMP2* in leaves, and *OsNRAMP3* is expressed in both tissues (Belouchi *et al*., 1997). In conditions of Fe excess, Quinet *et al*. (2012) noticed the repression of the gene *OsNRAMP1*, which is also involved in cadmium (Cd) accumulation (Takahashi *et al*., 2011a,b), while Finatto *et al*. (2015) observed the induction of another *NRAMP* gene, *OsNRAMP6*. *OsNRAMP5* is important not only for Fe, but also for manganese (Mn) and Cd transport (Ishimaru *et al*., 2012; Sasaki *et al*., 2012). *OsNRAMP3* is a vascular bundles-specific Mn transporter, showing once more that Mn commonly shares the same transporters with Fe in plants (Pittman, 2005; Cailliatte *et al*., 2010; Yang *et al*., 2013).

Inside the cell, Fe can be incorporated into proteins, stored in plastids and mitochondria, where it is found associated with ferritin (Duy *et al*., 2011; Vigani *et al*., 2013), or even in the vacuole of the cell (Gollhofer *et al*., 2014) (Figure 1). This compartmentalization can be useful for Fe homeostasis, especially in conditions of excess of this element. Ferritin is an iron storage protein that avoids damage caused by free radicals produced by the interaction iron/dioxygen (Goto *et al*., 1999). This protein has the capacity to store more than 4,500 Fe atoms in a soluble, non-toxic and bioavailable form (Briat and Lobreaux, 1997). In *Oryza glaberrima* S. and *O. sativa* the tolerance to Fe toxicity seems to be associated with ferritin synthesis (Majerus *et al*., 2007; Silveira *et al*., 2009). *A. thaliana* has four homologues of ferritin encoding genes (*AtFER1* to *AtFER4*), while in *O. sativa* two of these can be found (*OsFER1* and *OsFER2*) (Silveira *et al*., 2009). The Fe-dependent regulation of *AtFER1* and *ZmFER1* genes depends on the presence of a *cis*-element called Iron-dependent Regulatory Sequence (IDRS) in their promoter regions. The IDRS element is involved in the repression of FER genes in plants that grow under low concentrations of Fe (Petit *et al*., 2001). In case of Fe excess, the genes *OsFER1* and *OsFER2* show increased amounts of transcripts, with *OsFER2* being preferably upregulated (Stein *et al*., 2009). Similar results were found by Quinet *et al*. (2012), who also observed the induction of *OsFER1* and *OsFER2* genes in stress caused by excess of Fe in the soil. In other species, induction of *FER* genes by toxic amounts of Fe has also been observed.

Vacuoles are multifunctional organelles dynamically adjusted according to environmental conditions. This organelle has buffering capacity serving as a reservoir of metabolites, minerals, nutrients, and also as a deposit for toxic compounds, being crucial for the process of detoxification and for cellular homeostasis (Marty, 1999; Peng and Gong, 2014). The uptake of Fe by the vacuole is mediated by FERROPORTIN (FPN) (Morrissey *et al*., 2009) and by members of a family of VACUOLAR IRON TRANSPORTERS (VIT) (Zhang *et al*., 2012). In *A. thaliana* three homologues of *FPN* (*AtFPN1*/*AtIREG1*, *AtFPN2/AtIREG2* and *AtFPN3/AtIREG3*) were found (Curie and Briat, 2003; Morrissey and Guerinot, 2009; Merlot *et al*., 2014), while in *O. sativa* only two of these genes (*OsFPN1/OsFerroportin; OsFPN2/IREG3*) were detected (Bashir *et al*., 2011; Merlot *et al*., 2014). In Arabidopsis, iron accumulation in the vacuole of seed cells depends on *AtVIT1*. This protein is localized in the vacuolar membrane, and the gene is expressed in the developing embryo, seed and, in young seedlings, where the protein is predominantly associated with the vasculature (Kim *et al*., 2006). In rice the vacuolar membrane transporters encoded by *OsVIT1* and *OsVIT2* genes are involved in storage of iron in vacuoles of flag leaves, and the inhibition of these results in an increase of Fe in the seed, suggesting that new mechanisms are activated under this condition (Zhang *et al*., 2012), and under conditions of Fe excess, *OsVIT1* was increased (Finatto *et al*., 2015). In Arabidopsis, Fe remobilization from the vacuole to the cytoplasm is mediated by NRAMP3 and NRAMP4 (Peng and Gong, 2014).

## Quantitative Trait Loci

**Figure 3:**
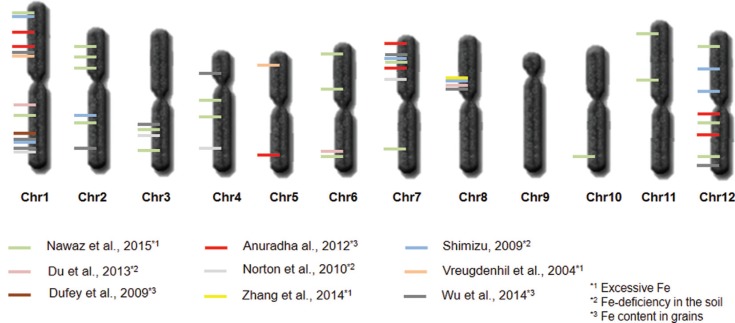
QTLs related to Fe metabolism. Map with the location of different QTLs related to tolerance to low and/or excessive amounts of Fe in the soil, and/or related to the variation of Fe content in grains.

In anaerobic conditions, high amounts of Fe^2+^ are taken up by plants, resulting in the accumulation of this element in the cell (Santos and de Oliveira, 2007). In rice, there is a differential response among cultivars to stress by Fe excess. When both susceptible and tolerant cultivars, BR-IRGA 409 and EPAGRI 108 respectively, were subjected to high concentrations of Fe there was less accumulation of this element and greater accumulation of ferritin in the tolerant cultivar, suggesting that this protein may be involved in this mechanism of tolerance (Silveira *et al*., 2009). However, a study by Panda *et al*. (2014) found that when there is Fe accumulation, the activity of aconitase and ferritin levels are higher in a cultivar that accumulates higher concentrations of Fe compared to the cultivar that has a lower concentration of this element. It is also interesting to highlight that a previous study showed that the accumulation of iron is not parallel to the level of ferritin expression in rice seeds overexpressing the *SoyFER* gene (of soybean ferritin), suggesting that Fe accumulation may be limited by the uptake and transport of this element (Qu *et al*., 2005). According to these studies, the mechanisms associated with tolerance to toxicity and accumulation of Fe are not well understood. However, studies related to the identification of Quantitative Trait Loci (QTLs) and genes whose products are responsible for the homeostasis of Fe and the accumulation of this mineral in the grain have been conducted (Figure 3 and Table S1), and the results of these surveys can assist breeding programs for toxicity tolerance, as well as biofortification for Fe content (Wu *et al*., 1998; Wan *et al*., 2003; Dufey *et al*., 2009; Shimizu, 2009; Wu *et al*., 2014). In this regard, three loci were identified on rice chromosomes 7, 8 and 9 that explain around 19-30% of the difference in the concentration of Fe in grains (Gregorio *et al*., 2000). Another study that did not analyze QTLs but gene expression, showed that higher concentrations of Fe in grains were positively correlated with the expression of the genes *OsYSL14*, *OsNAC5,* and negatively correlated with *OsNRAMP7*, *OsNRAMP8* and *OsFRO1* expression (Sperotto *et al*., 2010). On the other hand, *OsFER1*, *OsNRAMP4*, *OsNRAMP5*, *OsNRAMP6*, *OsYSL6*, *OsYSL12*, *OsYSL4*, *OsZIP8*, *OsZIP10* were correlated with higher concentration of Fe in grains. The functional characterization of these genes can help in getting biofortified rice genotypes with higher concentrations of Fe in grains. In a QTL analysis for tolerance to bronzing, using an F3 population from the cross between cv. Gimbozu (*japonica* genotype which is tolerant to Fe excess) and cv. Kasalath (*indica* genotype which is susceptible to Fe excess), seven QTLs associated with this feature were detected. These QTLs, which are located on chromosomes 1, 2, 7, 8 and 12, explain 99% of the phenotypic variation for bronzing and showed no detectable epistatic effect (Shimizu, 2009). In a population generated from the cross between cv. Azucena (tolerant *japonica*) and cv. IR64 (susceptible *indica*), a QTL on chromosome 1 was found associated with leaf bronzing index (Dufey *et al*., 2009). The association of this region with the bronzing index had already been detected earlier (Wan *et al*., 2003; Wu *et al*., 1998). Also in a QTL analysis in a population obtained from the cross between cv. Kasalath (susceptible *indica*) and cv. Koshihikari (tolerant *japonica*), a QTL on chromosome 3 was found associated with Fe concentration in the shoot (Fukuda *et al*., 2012). In a study conducted by Wu *et al*. (2014), populations from the crosses IR29 (susceptible *indica*) x Pokkali (tolerant *indica*) and Nipponbare (moderately tolerant *japonica*) x Kasalath (highly susceptible *japonica*) were used for identification of QTLs associated with tolerance to Fe excess. In the population IR29/Pokkali the authors identified seven QTLs for leaf bronzing, located on chromosomes 1, 2, 4, 7 and 12, explaining 9.2 to 18.7% of the phenotypic variation. In a Nipponbare/Kasalath/Nipponbare backcross inbred population, three QTLs were mapped on chromosomes 1, 3 and 8, and these QTLs explain 11.6 to 18.6% of the phenotypic variation. Additional studies demonstrated that the QTL on chromosome 1 was associated with shoot tolerance, and the QTL on chromosome 3 was associated with exclusion of Fe in roots. Similarly to the QTL studies for stress tolerance to Fe, much effort has been made in identifying QTLs associated with Fe content in grains. Four QTLs for Fe accumulation (*qFe1*, *qFe3*, *qFe4* and *qFe7*) located on chromosomes 1, 3, 4 and 7, accounting, respectively, for 16.2%, 21.4%, 9.7% and 15.5% of the phenotypic variation, were found in an F6 population from the cross cv. Bala (*indica*) x Azucena (*japonica*) (Norton *et al*., 2010). Using Composite Interval Mapping on an F6 population from the cross Madhukar x Swarna, it was possible to identify seven QTLs associated with iron accumulation (*qFe1.1*, *qFe1.2*, *qFe5.1*, *qFe7.1*, *qFe7.2*, *qFe12.1* e *qFe12.2*), which are located on chromosomes 1, 5, 7 and 12 (Anuradha *et al*., 2012). The candidate genes in these QTLs are: *OsYSL1* (LOC_Os01g13710), which is located within *qFe1.2*; *OsMTP1* (LOC_Os05g03780) located within *qFe5.1*; *OsNas3* (LOC_Os07g48980) located within *qFe7.1* and *qFe7.2*; *OsNRAMP1* (LOC_Os07g15460) located within *qFe7.2*; and *OsZIP8* (LOC_Os07g12890) located 0.3 Mb right of *qFe12.1*. Most phenotypic variance was explained by the QTL on chromosome 12 (71%) (Anuradha *et al*., 2012).

Mapping of a population derived from the cross Chunjiang 06 (*japonica*) x TN1 (*indica*) detected three QTLs for Fe accumulation in grains. The QTLs are located on chromosomes 1, 6 and 8, explaining, respectively 15.7, 10.6 and 22.3% of the phenotypic variation for Fe accumulation in grains (Du *et al*., 2013). A QTL related to Fe concentration, was detected on chromosome 8 through the study of a population from the cross cv. Lemont (*japonica*) x cv. TeQing (*indica*) (Zhang *et al*., 2014). A collection of Dale Bumpers National Rice Research Center of the USDA ARS, Stuttgart, AR, USA composed by 221 accesses of *O. sativa*, five accesses of *O. glaberrima*, two accesses of *Oryza rufipogon* Griff. and one of *Oryza nivara* Sharma et Shastry has been mapped aiming the identification of QTLs for different contents of minerals in the grain (Nawaz *et al*., 2015). In this study, the authors identified 11 genetic regions responsible for binding and transport of Fe, comprising the genes *OsZIP1* (Os01g0972200), *OsHMA4* (Os02g0196600), *OsACA2* (Os02g0176700), *OsZIP2* (Os03g0411800), *OsCNGC* (Os03g0758300), *OsZIP3* (Os04g0613000), *OsZIP5* (Os05g0472700), *OsZIP9* (Os05g0472400), *OsHma2* (Os06g0700700), Abc transporter (Os06g0607700), *OsNAS3* (Os07g0677300), Heavy metal transporter (Os07g0671400), Chy zinc finger (Os10g0456800) and *OsACA9* (Os12g0136900). In *A. thaliana*, two QTLs were identified on chromosomes 1 and 5, in a region in which genes (*ZIP10* and *NAS1*) are associated with Fe, playing a role in cation translocation (Vreugdenhil *et al*., 2004). Although further studies are required for the elucidation of mechanisms and genes related with the increase of iron concentration in seeds and stress tolerance for Fe excess, much work has already been developed in QTL mapping and its association with other metabolic pathways (Wan *et al*., 2003; Shimizu, 2009).

## Phylogeny

A phylogenetic study on members of gene families related to Fe homeostasis (*NAS*, *NRAMP, YSL, FRO* and *IRT)* was conducted in *O. sativa*, *A. thaliana*, *Physcomitrella patens* (Hedw.) Bruch & Schimp. and other monocots and dicots (Victoria *et al*., 2012). In this study, the authors found that *FRO* genes can be grouped into two clusters, but these do not separate monocots, dicots and bryophytes, a first clue indicating that the divergence of these genes occurred even before the diversification of land plants. Conversely, for *NAS* genes the formation of a group with monocots and dicots was observed. In the *IRT* family the genes were grouped into different clusters that separate monocots, dicots and bryophytes. For *NRAMP* genes, no evidence for divergence between groups of plants was observed, since genes from monocots and dicots were together in different clusters. Finally, the authors found that *YSL* genes possibly went through two duplication events, which probably occurred before the divergence of monocots and dicots (Victoria *et al*., 2012).

Phylogenetic analyses were also performed by Gross *et al*. (2003). In this study they analyzed a total of 43 genes belonging to five families: *YS*, *FRO*, *ZIP*, *NRAMP*, and ferritin proteins. The analysis of the *YS* family showed a relationship between predicted members of rice, Arabidopsis and maize (*Zea mays* L.), indicating that the putative new genes were homologous to maize *YS*, indicating that these may also have a role in Fe transport. The proteins from family FRO were separated from the burst oxidases, with a subdivision of FRO sequences, having *OsFRO1* in one group and *OsFRO2* in another. Members of the *ZIP* family were grouped in a single tree, with *OsZIP1* and *OsZIP6* more distantly related. The NRAMP family was divided into two classes, one more similar to *AtNRAMP1* and another to *AtNRAMP2*, in which the number of exons is determinative in grouping these sequences. The ferritin family showed a separation between each of the species analyzed, where mammalian ferritins were separated from their respective homologues. The separation was also noticed between monocots and dicots, and first we can observe the divergence of Arabidopsis genes, before genes from maize and rice diverge from each other.

## Strategies for Fe biofortification in rice

Biofortification is a process that increases the bioavailability of essential elements in the edible part of plants (White and Broadley, 2005; Zielinska-Dawidziak, 2015). Although Fe is the fourth most abundant element in the earth’s crust, little of this element is available for human nutrition by grains (Kim and Guerinot, 2007), a fact that contributes to ranking iron deficiency as the sixth risk factor for death and disability (WHO, 2015).

Although rice is a widely consumed food, it is not a rich source of iron, furthermore most of the Fe content of rice grains is accumulated in the aleurone and in the embryo, two parts that are lost during milling. After that, grains consist almost in its entirety of the endosperm, having lost up to 80% of the iron content and constituting a poor source of Fe for the human diet. This makes the evaluation of iron content in polished and unpolished grains an important piece of information when studying biofortification (Brinch-Pedersen *et al*., 2007; Paul *et al*., 2012; Bashir *et al*., 2013b).

Among plant breeding methods, transgenesis has high potential for Fe biofortification since this is a fast and efficient technique that is already being used for this purpose. Studies on rice biofortification by Fe using transgenesis were conducted using five different strategies. In the first strategy, the increase in the amount of Fe in the grain was achieved through the expression of soybean ferritin (*SoyFerh1*) under control of the *Glutelin* gene promoter from rice (*OsGLUB1*), which is specific for the endosperm. The higher expression of ferritin in the endosperm resulted in an at least two-fold increase of Fe in *japonica* cv. Kitaake (Goto *et al*., 1999) and in *japonica* cv. Taipei 309 (Lucca *et al*., 2001). The increase was 3.7-fold in *indica* cv. IR68144 (Vasconcelos *et al*., 2003) and 2.1-fold in *indica* cv. Pusa Sugandhi II (Paul *et al*., 2012).

In the second strategy, the increase in the amount of Fe in grains was due to the overexpression of genes involved in the synthesis of mugineic acid. When overexpressing Nicotianamine synthase (*NAS*) it was possible to notice an Fe content increase of even more than threefold in polished grains of the *japonica* cultivars Tsukinohikari (Masuda *et al*., 2009), Dongjin (Lee *et al*., 2009b), and Nipponbare (Johnson *et al*., 2011). When Dioxigenase (*IDS3*) was overexpressed it caused an Fe content increase of 1.4-fold in polished grains of the *japonica* rice Tsukinohikari (Masuda *et al*., 2008).

In the third strategy, the *OsYSL2* gene was inserted under the control of the promoter of *Sucrose transporter* (*OsSUT1*), resulting in increased expression of this gene in panicle and grains. This transformation increased by 4.4-fold the concentration of Fe in polished grains of the *japonica* cultivar Tsukinohikari (Ishimaru *et al*., 2010). The fourth strategy is a combination of the first three, generating the rice “*FER-NAS-YSL2*”, which presented a 4 to 6-fold increase in Fe content in polished grains of *japonica* cv. Tsukinohikari (Masuda *et al*., 2012), and a 3.4-fold increase in the other *japonica* cv. Paw Yin San (Aung *et al*., 2013).

Here it is interesting to note that Johnson *et al*. (2011) generated three populations of rice constitutively overexpressing *OsNAS1*, *OsNAS2* or *OsNAS3*. In this study nicotianamine, Fe and Zn concentrations were significantly increased in unpolished grains of all of these three overexpression populations, with the highest concentrations in the *OsNAS2* and *OsNAS3* overexpression populations.

Trijatmiko *et al*. (2016) evaluated polished grains of transgenic events grown in field conditions in two countries and showed that event NASFer-274 (containing *OsNAS2* and soybean ferritin (*SferH-1*) genes) showed good results without yield penalty or altered grain quality.

In the fifth strategy, besides increasing the Fe content in the grain, it was sought to increase tolerance to Fe deficiency as well. In this case, a concurrent insertion was used, with the *SoyFERH2* gene under the control of promoters of *OsGLUB1* and *OsGLB*, and also the *HvNAS1* genes Nicotianamine aminotransferase (*HvNAAT-A* and *HvNAAT-B*) and Mugineic acid synthase (*IDS3*) of barley, which encode enzymes for the biosynthesis of MAs. Here the transformed plants were tolerant to Fe deficiency and also capable of accumulating 2.5 to 4-fold of this mineral in polished grains (Masuda *et al*., 2013).

Also, the overexpression of the gene *OsIRT1* using a constitutive promoter (maize ubiquitin), resulted in higher concentration of iron and zinc in shoots and roots and an increase in tolerance to iron deficiency at the seedling stage. It was also possible to detect an increase in the concentration of these metals in mature grains, with 13% more iron and 12% more zinc (Lee and An, 2009).

Similar data were found in plants overexpressing *OsIRO2*. These plants were shown to be more tolerant to iron deficiency and presented an increase in Fe content in shoots (two-fold increase) and grains (more than twice) when grown in calcareous soil (Ogo *et al*., 2011).

In addition, another strategy used is the knockdown of the gene *OsVIT2*, an important gene in the increase of iron concentration (Zhang *et al*., 2012). Bashir *et al*. (2013b) showed that transgenic *OsVIT2-knockdown* plants had an increase (1.8-fold) in the concentration of iron in polished grains. This suggests that the disruption of this gene helps in increasing the amount of iron in the grains, constituting a possible strategy for producing biofortified rice.

Although the strategies using transgenesis resulted in an increase in Fe content in grains, it is known that the polishing process is still responsible for major losses of this mineral. However, we should not forget that, the location of Fe in the grain may vary according to genotype (Doesthale *et al*., 1979; Sperotto *et al*., 2010). Thus, further studies should be conducted aiming to develop new strategies for internalization of Fe (Sperotto, 2013).

The flag leaves are the main source of photoassimilates for the development of seeds in rice. The Fe concentration of the flag leaf decreases during the reproductive development in rice, whereas the iron content of the grains increases. An interesting fact is that cultivars with lower Fe accumulation in grains show higher Fe accumulation in flag leaves. This was demonstrated in a study that showed that there is an iron remobilization from the flag leaves to the grains, and increasing this remobilization can help in obtaining biofortified grains (Sperotto *et al*., 2010). Still it is interesting to remember that other studies conducted by the same group showed that flag leaf removal (at anthesis) under field conditions did not affect seed Fe and Zn accumulation, suggesting that the flag leaves can be important, but not necessary, unless under low iron supply from the soil (Sperotto *et al*., 2013; Sperotto, 2013).

The commercialization of genetically modified Fe biofortified crops has some limitations, either by farmers (changes in the appearance of the product) and consumers (high cost and acceptance of genetically modified organisms). In this sense, methods based on the selection of genotypes that are rich in Fe, followed by hybridization, may be better accepted (Zielinska-Dawidziak, 2015).

Rice Germplasm banks can be screened to identify genotypes that can absorb and store Fe more efficiently, so more QTLs related to these characteristics can be mapped and introgressed in elite varieties. In this case, one needs to take into account the natural variation that occurred during evolution, taking advantage of the effects of specific interactions between different genes and alleles (Schuler and Bauer, 2012; Pinson *et al*., 2015). An example of the potential for exploitation of these banks is the 4-fold difference found when comparing the iron content of aromatic and traditional varieties (Mulualem, 2015).

The natural variation related to Fe accumulation in rice grains that was already detected is quite low. In addition, grinding and polishing the grains results in a loss of up to 80% of this element (Brinch-Pedersen *et al*., 2007). Furthermore, the Fe concentration is deeply influenced by the interaction between genotype and environment (Graham *et al*., 1999). However, despite these limitations, the International Rice Research Institute (IRRI) has developed the cultivar IR68144, which has about twice the concentration of Fe when compared with local varieties used in the Philippines (Gregorio *et al*., 2000).

The development of cultivars with increased iron content in the grains, even at relatively low levels, associated with results of the characterization of 1,138 genotypes, that identified a variation of 6.3 to 24.4 μg.g^-1^ of Fe in grains, suggests that there is genetic potential for the development of other, new varieties with high accumulation of Fe (Gregorio *et al*., 2000; Mulualem, 2015). Furthermore, the genetic variability for the content of phytic acid can also be exploited, and these possibilities make the future of genetic progress seem really optimistic (Liu, 2005).

## Conclusions

Being essential in the composition of different proteins and metabolic pathways, iron is vital for animal and plant health. Actually, it is an element capable of generating toxic effects due to its high bioavailability and is also a problem due to its low availability. To solve this problem, studies aiming the identification and understanding of pathways related to the regulation of iron metabolism are being conducted, combined with molecular markers in the identification of QTLs associated with these pathways. Furthermore, phylogeny can be used to better understand the evolution of the involved genes aiming not only to decrease the sensitivity of rice both to the lack and to the excess of iron in the soil, but also to help in the generation of biofortified plants with higher iron content in the grains.

Looking at these studies it is possible to see success, not only in the description of regulatory pathways, but also in breeding for improved varieties. Advances continue to be made and obstacles being overcome. In the future we should add efforts towards identifying more QTLs related to iron excess tolerance, and to increase iron content in grains. This, allied to the exploration of the existing variation for genes that have proven to be important in experiments involving transgenic analysis, should enable us to achieve greater market acceptance and to reduce bureaucratic obstacles, which greatly hinder the release of genetically modified organisms.

Although the genetic progress may seem difficult at certain times, our ability to deal with iron metabolism in rice has increased, and soon we should obtain cultivars that will be highly tolerant to iron stress, both against excess and lack of this mineral, and, allied to this, we should also be able to develop biofortified plants with higher content of iron in their grains, helping in the fight against anemia and providing better quality of life to humanity.

## Supplementary Material

The following online material is available for this article:

Table S1 - Positions of the QTLs related to Fe metabolism shown in Figure 3.
