# Supplementary material for: Dealing with iron metabolism in rice: from breeding for stress tolerance to biofortification
Source: Genet Mol Biol. 2017 Mar 16;40(1 Suppl 1):312–25. doi: 10.1590/1678-4685-GMB-2016-0036 (PMC5452141; doi:10.1590/1678-4685-GMB-2016-0036)
Supplement: Supplementary file 1 [file 1415-4757-gmb-1678-4685-GMB-2016-0036-Suppl01.pdf]

Table S1 - Positions of the QTLs related to Fe metabolism shown in Figure 3.

Characteristic	Chromosome[a]	Marker[b]	LOD	Additive effect[c]	Details	References
**Fe accumulation in rice grains**	1	RM246-RM5461	3,160	-1,6312	Explained variance (%): 15.7	Du et al., 2013
	1	RM431			Gene and locus: *OSZIP1* Os01g0972200	Nawaz et al., 2015
	1	AD121	2,71		Position (cM): 41.5; Explained variance (%): 6.4	Vreugdenhil et al., 2004
	1	RM243–RM488	21,9	−59.05	R^2(%)[d]: 69	Anuradha et al., 2012
	1	RM488–RM490	21,9	−59.04	R^2(%)[d]: 69.2	Anuradha et al., 2012
	1	C949+0	5,1		R^2(%)[d]: 16.2	Norton et al., 2010
	2	RM145, RM555 and RM279			Gene and locus: 1. *OsHMA4* Os02g0196600; 2. Aspartate aminotransferase	Nawaz et al., 2015
	2	RM279 and RM555			Gene and locus: *OsACA2* Os02g0176700	Nawaz et al., 2015
	2	BH144	2,96		Position (cM): 9.6; Explained variance (%): 7.3	Vreugdenhil et al., 2004
	3	MRG4864/ RM5864 and RM3400			Gene and locus: *OsZIP2* Os03g0411800	Nawaz et al., 2015
	3	RM8203			Gene and locus: *OsCNGC14* Os03g0758300	Nawaz et al., 2015
	3	R1618-7	4,5		R^2(%)[d]: 21.4	Norton et al., 2010
	4	RM3217			Gene and locus: *OsZIP3* Os04g0613000	Nawaz et al., 2015
	4	RM349+0	3,4		R^2(%)[d]: 9.7	Norton et al., 2010
	5	RM161			Gene and locus: 1. *OsZIP5* Os05g0472700; 2. *OsZIP9* Os05g0472400	Nawaz et al., 2015
	5	RM574–RM122	25,3	−59.1	R^2(%)[d]: 69.2	Anuradha et al., 2012
	6	RM340-RM494	2,05	-1,3498	Explained variance (%): 10.6	Du et al., 2013
	6	RM162			Gene and locus: 1. *OsHMA2* Os06g0700700; 2. ABC transporter domain containing protein Os06g0607700	Nawaz et al., 2015
	7	RM1335			Gene and locus: 1. *OsNAS3* Os07g0689600; 2. Peroxidase Os07g0677300; 3. Potassium transporter 7 *OsHAK7* Os07g0669700; 4. Heavy metal transporter Os07g0671400	Nawaz et al., 2015
	7	RM234–RM248	27,9	59,1	R^2(%)[d]: 69	Anuradha et al., 2012
	7	RM248–RM8007	27,2	59,1	R^2(%)[d]: 69	Anuradha et al., 2012
	7	R1440+0	4,9		R^2(%)[d]: 15.5	Norton et al., 2010
	8	RM4085-RM1111	4,14	1,9368	Explained variance (%): 22.3	Du et al., 2013

Characteristic	Chromosome[a]	Marker[b]	LOD	Additive effect[c]	Details	References
Tolerance to Fe toxicity	10	RM271			Gene and locus: Os10g0456800 CHY zinc finger family protein	Nawaz et al., 2015
	12	RM19			Gene and locus: *OsACA9* Os12g0136900	Nawaz et al., 2015
	12	RM17–RM260	33,8	−59.5	R^2(%)[d]: 71	Anuradha et al., 2012
	12	RM 260–RM7102	33,4	−59.5	R^2(%)[d]: 71	Anuradha et al., 2012
	1	STSI16-RM220				Shimizu, 2009
	1	RG220B-RG810				Shimizu, 2009
	1	id1008684		0,82	R^2(%)[d]: 10.6	Wu et al., 2014
	1	id1021920		−0.72	R^2(%)[d]: 12.7	Wu et al., 2014
	1	R2417+0	4,4		R^2(%)[d]: 12.4	Norton et al., 2010
	1	RM034–RM246	7,62	-0,61	R^2(%)[d]: 18.5	Dufey et al.,2009
	1	RM443–RM403	7,92	-0,67	R^2(%)[d]: 18.1	Dufey et al.,2009
	1	RM265–RM315	3,78	0,4	R^2(%)[d]: 8.1	Dufey et al.,2009
	2	RM221-RM6				Shimizu, 2009
	2	id2013434		0,94	R^2(%)[d]: 10.3	Wu et al., 2014
	2	RM324–RM550	8,53	-1,05	R^2(%)[d]: 35.7	Dufey et al.,2009
	3	RG409+2	3,2		R^2(%)[d]: 10.6	Norton et al., 2010
	4	id4002852		0,85	R^2(%)[d]: 18.7	Wu et al., 2014
	4	id4005867		0,69	R^2(%)[d]: 9.9	Wu et al., 2014
	6	RZ516+0	3,4		R^2(%)[d]: 10.4	Norton et al., 2010
	7	RC-RM11				Shimizu, 2009
	7	id7000519		1,11	R^2(%)[d]: 11.7	Wu et al., 2014
	8	RM44-RM42				Shimizu, 2009
	11	RM254–RM224	3,74	-0,36	R^2(%)[d]: 10.5	Dufey et al.,2009
	11	RM224–RM144	3,75	-0,31	R^2(%)[d]: 7.5	Dufey et al.,2009
	11	RM004b–RM019	5,24	0,75	R^2(%)[d]: 17.6	Dufey et al.,2009
	12	id12010050		0,96	R^2(%)[d]: 10.6	Wu et al., 2014

a Chromosome number where the QTL were detected
b Marker in which is located the most probable position of the QTL
c Effect of substituting a single allele from one parent to another. Positive value indicates that the allele contributes to increase the value of the parameter and negative value indicates that the allele contributes to increase the value of the parameter
d Part of the phenotypic variation explained by the QTL (%)
LOD: likelihood ratio

References for Table S1:

Anuradha K, Agarwal S, Rao YV, Rao KV, Viraktamath BC and Sarla N (2012) Mapping QTLs and candidate genes for iron and zinc concentrations in unpolished rice of Madhukar×Swarna RILs. Gene 508:233–240.

Du J, Zeng D, Wang B, Qian Q, Zheng S and Ling HQ (2013) Environmental effects on mineral accumulation in rice grains and identification of ecological specific QTLs. Environ Geochem Health 35:161–170.

Dufey I, Hakizimana P, Draye X, Lutts S and Bertin P (2009) QTL mapping for biomass and physiological parameters linked to resistance mechanisms to ferrous iron toxicity in rice. Euphytica 167:143–160.

Nawaz Z, Kakar KU, Li X, Li S, Zhang B, Shou H and Shu Q (2015) Genome-wide association mapping of Quantitative Trait Loci (QTLs) for contents of eight elements in Brown Rice (*Oryza sativa* L.). J Agric Food Chem 63:8008–

8016.
Norton GJ, Deacon CM, Xiong L, Huang S, Meharg AA, Price AH (2010) Genetic mapping of the rice ionome in leaves and grain: Identification of QTLs for 17 elements including arsenic, cadmium, iron and selenium. Plant Soil 329:139–153.
Shimizu A (2009) QTL analysis of genetic tolerance to iron toxicity in rice (*Oryza sativa* L.) by quantification of bronzing bcore. J New Seeds 10:171–179.
Vreugdenhil D, Aarts MGM, Koornneef M, Nelissen H and Ernst WHO (2004) Natural variation and QTL analysis for cationic mineral content in seeds of *Arabidopsis thaliana*. Plant Cell Environ 27:828–839.
Wu L, Shhadi MY, Gregorio G, Matthus E, Becker M and Frei M (2014) Genetic and physiological analysis of tolerance to acute iron toxicity in rice. Rice 7:8.
